# Sensationalist social media usage by doctors and dentists during Covid-19

**DOI:** 10.1177/20552076211028034

**Published:** 2021-06-29

**Authors:** Richard WM Law, Shalini Kanagasingam, Kartina A Choong

**Affiliations:** 1 Department of Acute Medicine and Urgent Care, Tameside & Glossop Integrated Care NHS Foundation Trust, Ashton-under-Lyne, UK; 2 School of Dentistry, 6723 University of Central Lancashire, University of Central Lancashire, Preston, UK; 3 School of Justice, 6723 University of Central Lancashire, University of Central Lancashire, Preston, UK

**Keywords:** Covid-19, social media, e-professionalism, doctors, dentists, GMC, GDC

## Abstract

**Introduction:**

Many doctors and dentists took to social media to raise alarm and/or express professional opinion, dissatisfaction, anger and/or incredulity associated with the Covid-19 pandemic. Although most of these social media posts involved practitioners from abroad, this article explores whether they would attract fitness to practise investigations had they been posted by UK-based medical and dental practitioners. In particular, it asks whether such conduct comes into conflict with the existing professional standards issued by the General Medical Council (GMC) and the General Dental Council (GDC). It questions also whether those guidelines should be updated and/or further clarified in view of the extraordinary circumstances posed by the pandemic.

**Method:**

An exploratory study was conducted using sensationalist pandemic-related social media posts by doctors and dentists discovered during the first half of 2020 (n = 11). The contents were analysed qualitatively using documentary analysis using coding terms based on the professional standards on social media published by both the GMC and the GDC. The codes generated common and recurring themes that were used to structure discussion.

**Findings:**

This study provides a partial insight as to the likely motivations of doctors and dentists to use social media in a manner that may not necessarily lend well to the professional standards expected. In a majority of instances, doctors and dentists who posted social media material with a sensationalist outlook tended to focus on single-issue campaigns pertaining to specific aspects of the Covid-19 pandemic. These issues included controversial commentary on acute shortages of personal protective equipment and attendant occupational risks to clinical staff to Covid-19 infection; criticisms directed towards regulatory bodies in the handling of the pandemic; and professional advice to the general public which was later found to be inaccurate.

**Conclusions:**

Social media offer opportunities for healthcare professionals to play a constructive role in raising awareness, disseminating information, and promoting solidarity in the management of the Covid-19 pandemic. However, doctors and dentists must carefully consider the ethical and professional pitfalls involved in sensationalist social media posts. The GMC and the GDC should, at the same time, regularly update and clarify their social media guidance in response to major global events like a pandemic as well as advances in social media technology.

## Introduction

Social media platforms have been useful in helping people maintain connectivity during the Covid-19 pandemic.^1^ With so much of what constituted normal life fractured by measures like social distancing, working from home, lockdown, self-isolation and travel restrictions, it was reported that more than half of the world’s population (i.e. 3.96 billion), were active social media users by the middle of 2020.^2^ Defined as web-based applications which facilitate public sharing of information between individuals as well as within vested groups or professional communities,^3^ these platforms provide numerous opportunities for social and professional networking, media sharing, blogging and online content production.^4^ Communication via social media is heavily reliant on user engagement, the technical features of the social media platform, and the relationships developed by social interactions online. Studies have shown that increased social media engagement requires the influence of a ‘critical mass’ of individual users^5,6^ and that sentiments from this critical mass group of individuals may drive others within the user group to create material to reflect those views and opinions.^7,8^ From a social media engagement perspective, the audience interaction may influence, inform, and drive individual contribution online. In other words, feedback from a group of individuals within a social media following may motivate the author to produce social media material to promote the overall views of that group. Therefore, in an attempt to garner audience support, create social solidarity, and expand endorsement, authors may opt for a sensationalist standpoint to put forward their respective arguments. Indeed, news which could shock and elicit a strong emotional reaction are known to attract a wide audience.^9^

The surge of social media engagement during the pandemic was also discernible among doctors and dentists.^10^ Many from these two professional groups have been increasingly reliant on social media to keep pace with evolving best practice and effective dissemination of new knowledge on the novel coronavirus SARS-CoV-2. With practitioners seeking specialised social media groups as a means to share professional advice to queries in real time, Facebook groups, for instance, have emerged with tens of thousands of members worldwide, all sharing comparative technical information and updates.^11^ Thus, mass sharing of information via social media may facilitate adoption of proven treatments whilst simultaneously identifying potential efficacious treatments. Furthermore, social media can be useful for conveying health messages to the public.^12^ Similarly, with didactic training less practicable, medical and dental professionals have sought social media platforms to help support clinical educational needs and development. Although the use of social media in health education has already been broadly acknowledged in the extant literature,^13^ the pandemic has accentuated this phenomenon.^14^ However, given the scale of change that has taken place in such a short period of time,^15^ a number of medical and dental practitioners have also resorted to platforms like Facebook, Twitter, YouTube and blogs for the purposes of publicly expressing their professional views, concerns, dissatisfaction, anger, and/or incredulity. Such social media usage could be deemed ‘sensationalist’ especially if the online posts were presented in a manner intended to cause shock or evoke strong emotional reactions from fellow professionals and/or from the lay readership.

Like the general public, medical and dental practitioners are expected to abide by the law when sharing posts online.^16^ However, they are usually subject to an additional layer of regulation from their statutory professional regulatory bodies, which in the United Kingdom (UK) are the General Medical Council (GMC)^17^ and the General Dental Council (GDC).^18^ Both councils have issued their respective social media guidelines in the last decade, in response to increased social media usage amongst their registrants. They emphasised the importance of maintaining the same professional behaviour and standards as would be expected in traditional face-to-face encounters. In other words, there is an expectation that professional conduct online is at parity with that of offline.^19^ They both adopted the position that the failure to follow published guidance in relation to social media may incur a formal investigation and where necessary, disciplinary action.^20,21^ The various broad themes identified from the GMC and GDC social media guidelines are summarised in Table 1 below.

**Table 1.** A comparison of broad themes in the GMC and GDC social media guidance.

Theme	GMC	GDC
Confidentiality	Doctors must not discuss patients and their care via publicly accessible social media platforms.	Anonymised patient information can be shared, with the patient’s explicit consent.
Maintaining boundaries	Warned of risks faced if social and professional boundaries become unclear. If a patient contacts a doctor via their private profile on social media, they should be directed to their professional profile where appropriate.	The dental team are advised to consider carefully before accepting ‘friend requests’ from patients.
Respect for colleagues and profession	Doctors are expected to be fair and respectful to all colleagues in their social media interactions. Attention is drawn to the laws covering copyright and defamation which also apply to online posts. This should be kept in mind when commenting on individuals and organisations in both personal and professional capacities.	Dental professionals should not post any information which may compromise public confidence in their dental professionalism. The expectation is to be respectful and fair to all colleagues and must not bully, harass or discriminate against them in all forms of communication including on social media. Dentists can be held responsible if caught sharing content of this nature, even if they did not create the offensive post.
Raising concern	Not explicitly addressed.	Explicitly advises against using social media for raising concerns. Dental professionals are expected to adhere to their workplace whistleblowing procedures and can refer to the GDC’s official guidelines for raising concerns relating to dental professionals.
Anonymity	Should doctors decide to identify themselves as medical professionals on social media, they are expected to identify themselves by name. This is important as information posted by a doctor may be trusted by the public and could also be taken as representing the stance of the medical profession.Doctors are warned of the need to be careful when posting anonymously as this can be traced back to the source.	It is implied that dental professionals may choose to post under a pseudonym. However, posting under a different username will not guarantee concealment of one’s identity. Privacy settings should be regularly reviewed, although there is always a risk that posts can be copied and redistributed.
Conflict of interest	Doctors should always be transparent about any conflict of interest and declare any financial interests linked to their posts.	Not explicitly addressed.

These guidelines make clear that doctors and dentists are expected to be more circumspect and restrained when interacting on online platforms when compared to the general public. This article sets out to explore whether the sensationalist online posts from doctors and dentists relating to Covid-19 would have contravened the GMC and the GDC guidelines had they been engaged in by UK-based medical and dental practitioners. From there, it considers whether the existing GMC and GDC guidance should be updated and/or further clarified in view of the extraordinary circumstances posed by the pandemic.

## Methodology

To address the questions raised in this article, a documentary analysis was undertaken, and the findings were then examined against the social media guidelines issued by the GMC and GDC. Documentary analysis has been successfully utilised in the field of digital healthcare research and in health policy research.^22^ It is a systematic procedure for reviewing or evaluating a wide variety of documents (including social media posts), and the data generated is analysed, interpreted and organised into themes through content analysis. Given that social media content are often created in the public domain and freely available without authors’ consent, document analysis is a relevant and effective research tool for data selection in a non-reactive and unobtrusive manner.^23^

Due to the professional interests of the team, we had discovered 11 sensationalist social media posts by doctors and dentists relating to the pandemic during the first half of 2020. These social media materials were publicly available on well-known platforms such as Facebook, Twitter, and YouTube. We decided to conduct an exploratory study using these as examples. Given the small number, each of the social media posts was reviewed manually, rather than using qualitative analysis software. They were coded on the basis of the GMC and GDC professional standards on the use of social media. These codes generated themes which were used to structure discussion.

## Findings

The findings of the documentary analysis are presented in Table 2 below. There were three major themes which emerged from the documentary analysis. These are: whistleblowing; criticism of regulatory bodies; and provision of inaccurate information.

**Table 2.** Characteristics and the coding of the social media posts.

Case	Author(s) country of origin	Date	Social media platform	Covid-19 context	Brief summary of argument(s) and relevant quotes	Motivations	Repercussions following social media activity	Target audience	Relevant GMC and GDC professional standards potentially broached (Themes)
1	@BlankeBedenken “Naked Qualms”Medical Professionals from Germany	22/04/20	Twitter	Shortages of personal protective equipment (PPE) and inherent risks of Covid-19 infection	Lack of PPE for frontline healthcare professionals during Covid-19 is analogous to the vulnerability of being naked without clothing“*But the protective clothing, disinfectant and single-use masks were soon not to be had. Despite their concerns about them and their patients being insufficiently protected against contracting the virus, across the country GPs and their teams are caring for people*.”	Protest in the nudeDemand for PPERaise awareness of lack thereof	None known	Healthcare professionals General public	Whistleblowing
2	Dr Alain ColombiéGeneral Practitioner from France	22/03/20	Facebook	Shortages of PPERisks to doctors in treating patients with Covid-19 with inadequate PPE	Author denounces a “guilty” lack of preparation for the pandemicInadequate PPE akin to being “*cannon fodder*”	Protest in the nudeRaise awareness on the plight of healthcare workersAppeal to the Office of the French President	Escalation of complaints already made (by the author) against local authorities in relation to distribution of PPE	Healthcare professionalsGeneral publicLeaders of local and national Governments	Whistleblowing
3	#DentisteapoilDental Professionals from France	25/04/20	Twitter	Shortages of PPEOccupational risks to dental professionals	Authors denounce the French Authorities for forcing dentists to work without adequate PPE.Vulnerability to Covid-19 is analogous to being “*naked*” without clothing.Dentistry – A high risk profession during Covid-19 “*Around the world, dentists are required to see emergencies but are at the bottom of the priority lists for PPE, endangering their health and those of their patients*” (translated from French).	Protest in the nudeRaise awareness on the plight of dentistsAppeal to the French Government	None known	Dental professionalsGeneral publicLeaders of local and national governments	Whistleblowing
4	Dr Ming Lin Emergency Department Physician from USA	25/03/20	Facebook	Patient safety in Emergency DepartmentLack of coronavirus safety precautionsShortages of PPEDelays in Covid-19 test resultsConcerns over Covid-19 screen practices in waiting rooms.	Author describes inadequacies to workplace Covid-19 safety precautionsOccupational risksRisks to patient safetyFactors pertaining to poor workplace planning “*Shame on Peacehealth*”	Raise awareness over potentially unsafe workplace practicesRaise awareness over hospital planning during the pandemic.	The author’s workplace contract was terminated following the Facebook posting.	Healthcare professionalsGeneral publicAppeal to workplace management	Whistleblowing
5	Dr Hany BakrOphthalmologist in Egypt	10/04/20	Facebook	Shortages of PPEDistribution of medical aid	Author criticises the Egyptian authorities via Facebook for their decision to send aid to Italy and China to the detriment of their home country at the start of the pandemic	Raise awareness of governmental public health policies	Author was arrested on charges based on misusing social media and spreading false news	General public	Whistleblowing
6	@DentistGoneBaddDental Professional in UK	06/06/20	General Dental Practitioner UK (GDPUK) Blog	Leadership of the UK dental regulatory body during the pandemicThe lack thereof contributed to the poor morale of dentistsLack of financial support to dentists who have lost work during the pandemicPatient safety	Author criticises the competence of the GDC on its response to the Covid-19 pandemicLack of leadership at the organisation“*I don’t trust the GDC’s judgement as a regulatory body, I have sound reason to question its veracity, competence, and I cast much doubt on its sincerity, powers of reasoning and grasp on economics. They also obfuscate like they are the Olympic champions. Most of all, I don’t trust them with money*.”	Raise awareness of the need for organisational change within the regulatory body	None known	Dental professionals	Criticising regulatory body and its personnel
7	Dr Dan EricksonDr Artin Massihi Urgent Care Physicians Bakersfied, USA	22/04/20	YouTubeTwitter	Covid-19 pandemic – questionable significanceCovid-19 lockdown restrictions unnecessary	Authors argue that the coronavirus was similar to influenza and that the likely death rates from the Covid-19 pandemic is not too dissimilar to seasonal influenza outbreaksDeath rates from the coronavirus are low and therefore the public need not observe Covid-19 lockdown restrictionsThe spread of coronavirus is not dangerous“*We understand microbiology. We understand immunology and we want strong immune systems. I don’t want to hide in my home, develop a weak immune system and then come out and get a disease*.”	Provide a different perspective to mainstream opinionPromote the authors’ chain of urgent care centres	Authors criticised by the American College of Emergency Physicians and the American Academy of Emergency MedicineCriticisms from the local health authorities and Senate Health Committee	General public	Inaccurate or unfounded professional opinion
8	Dr Muhammad Iqbal Adil Consultant Surgeon from the UK	24/04/20	YouTubeTwitterFacebook	Covid-19 is a manufactured hoax	Novel coronavirus had been*“[o]rchestrated by the elite in order to control the world”“[w]e have come to the conclusion that the Coronavirus is actually a hoax”*	Dispel current Covid-19 scientific and clinical narrative and practices	The General Medical Council on 1 June 2020 imposed immediate suspension on the author’s licence to practise following this social media activity	General public	Inaccurate or unfounded professional opinion
9	Didier RaoultClinical Microbiologist from France	28/02/20	TwitterFacebookYouTube	Covid-19 treatment proposed	Based upon his clinical experience and recent research findings, advocates the efficacy of hydroxychloroquine as a treatment for Covid-19	Advances research findingsPublicises expertiseIncreases public awareness of the availability of potential treatment	Research findings later found to be unsubstantiatedTreatment was later found by high profile clinical trials to be ineffective against Covid-19 infections	General publicHealthcare professionals	Inaccurate or unfounded professional opinion
10	Dr Jeffrey VanWingenPrimary Care Doctor from USA	24/03/20	YouTube	The washing of fresh fruit, vegetables, and groceries with soap as a means to mitigate risks of coronavirus infection	Advises the public to keep new groceries and fresh produce in a garage or porch for 72 hours before use; food containers bought at store to be discarded or disinfected.	Publicises expertiseIncreases public awareness that coronavirus is transmissible via surfaces.	Currently no evidence that food or food packaging are vectors for coronavirus infectionAdverse effects of detergents and soap on ingestion when used to wash fresh produce	General public	Inaccurate or unfounded professional opinion
11	Dr Brian McDigi	07/06/20	Change.org	Petition to disband or reform the GDC and fire the Chief Dental Officer due to failures of support and guidance to the dental profession at the onset of the Covid-19 pandemic	The regulator and the Chief Dental Officer of England had failed to provide timely and effective advice to dentists with regard to changes to practice during the pandemicRepeated failures in leadership and poor communication with the wider dental profession.Dissatisfaction with lack of concessions to annual retention fee as incomes of registrants fell during the pandemic“*The dental profession is utterly ashamed of our regulator and the Chief Dental Officer who has provided a consistently confusing and lacking response to leading Dentistry in the guidance of dentists in treating the public*.”	Petition the UK government to implement changes to the regulatory body and install a new Chief Dental Officer (England)	None known	General public Professional peersUK government	Criticising regulatory body and its personnel

In relation to the first theme, cases 1 to 3 show that several doctors in Germany^24^ and several dentists in France,^25^ have staged protests in the nude on social media to raise public awareness of the inherent risks to medical and dental professionals working with inadequate personal protective equipment (PPE) in their respective countries. They have likened their vulnerability to that of being naked without clothing: *“the protective clothing, disinfectant and single-use masks were soon not to be had”* (case 1), thus *“endangering their health and those of their patients”* (case 3). A clinician compared frontline healthcare staff to *“cannon fodder”* (case 2) whilst another remarked *“I was trained to sew up wounds, why am I now having to sew my own face mask?”* (case 1). Less strikingly, but no less attention-grabbing, case 4 shows the action of a doctor in the USA who through his Facebook profile, exposed the policies of his employing organisation in relation to the lack of PPE, unacceptable delays in receiving coronavirus test results and risky virus screening practices.^26^ Similarly, some doctors in Egypt (case 5) have used social media to draw attention to the poor working conditions during the early days of the pandemic.^27^ Issues raised included the acute shortage of hospital beds, lack of emergency medications, and the unavailability of Covid-19 testing kits.

The second relates to the usage of social media as an outlet to produce controversial observations and a forum to hold regulatory bodies and their relevant personnel to account. To this effect, a former National Health Service (NHS) dentist had actively mocked the GDC on GDPUK. This is a UK-based site for dentists and dental professionals to discuss all aspects of their professional work and is popular amongst dental industry advertisers. Although the majority of the contents are ‘closed group’ and are restricted to dental professionals via registration, some contents are open access to the general public. Posting under a pseudonym (case 6), the dentist criticised the regulatory body for ignoring patient safety issues and paying little regard to the financial hardship of its registrants during the pandemic: *“I don’t trust the GDC’s judgement as a regulatory body, I have sound reason to question its veracity, competence, and I cast much doubt on its sincerity, powers of reasoning and grasp on economics.”* Though deliberately written in a tongue-in-cheek manner, the underlying accusations are serious as they cast doubt on the integrity of the GDC.^28^ Some dentists also started an online petition to call for the disbandment or reform of the GDC as well as the dismissal of the Chief Dental Officer of England (case 11).^29,30^ Utilising social media as a means to promulgate discontent amongst dental professionals, they proclaimed that *“[t]he dental profession is utterly ashamed of our regulator and the Chief Dental Officer who has provided a consistently confusing and lacking response to leading Dentistry in the guidance of dentists in treating the public.”*

Thirdly, clinicians appear to perceive themselves free to express their views in their own individual style. Some clinicians have presented erroneous information and/or downplayed the seriousness of Covid-19 on social media. Two doctors from Bakersfield USA (case 7), through their YouTube broadcast, suggested that the coronavirus was *“nothing but flu [influenza] and that is not serious”* and as such there is no need to observe lockdown restrictions.^31^ They proceeded to present clinical data, which was later disputed, to further their suggestion that Covid-19 was not dangerous. Similarly a consultant surgeon in the UK with over 30 years of clinical experience (case 8), claimed that the novel coronavirus had been *‘[o]rchestrated by the elite in order to control the world”*.^32^ This specific claim was made across various social media platforms and online interviews with the effect to portray Covid-19 as nothing but a *“manufactured hoax”*. The GMC subsequently moved to impose a suspension order on the surgeon pending formal investigation. Another controversial view came from France, where a doctor had advocated via a Twitter account the efficacy of hydroxychloroquine as a treatment for Covid-19 infections (case 9).^33^ This was based on data that was not peer reviewed, and which clinical trials subsequently reported as having no clinical benefit to hospitalised patients with Covid-19 infections.^34^ In addition to controversy, some clinicians have conflated their views with poorly qualified expertise. For example, a USA-based physician, through his YouTube broadcasts (case 10),^35^ gave factually inaccurate guidance on the handling of food groceries in relation to Covid-19.^36,37^ The social media platforms utilised by those clinicians to generate their online content were broadly reflective of the popularity of the various social media platforms amongst the general population.^38^

## Discussion

As observed above, social media have been used to express concerns, frustrations and unconventional opinions that were pertinent to the Covid-19 pandemic. Some individuals within the medical and dental professions have used the platforms as an arena for whistleblowing in bold and unusual ways to draw attention to, among other things, shortages of personal protective equipment (PPE) and their individual well-being. Some have expressed strong criticisms over their regulators’ management of the pandemic. Meanwhile others have created materials to express professional opinions relating to the novel coronavirus which were later found to be inaccurate or unfounded. Thus, how would the GMC and the GDC respond to such controversial social media contributions had all the posts originated from UK-based medical and dental practitioners?

Before this question is addressed, it is necessary to remark that sensationalist social media contributions from doctors and dentists are not a new phenomenon. These have previously been reported on issues pertaining to religion, politics, and on discourses around demographics and society in general.^39^ Social media contents created by healthcare professionals using a somewhat controversial approach is not without its risks. This study has found, that in the cases identified, there were significant reactions that had ranged from reverberations of social support to repercussions that involved professional sanctions and regulatory interventions. In all the social media posts analysed, the likely target audience and consumers of these contributions seemed to be the general public more so than professional peers. Social media can be an effective tool for raising awareness for the average person, who in all likelihood would have no access to conventional news or advertising media. For example, social media had enabled healthcare professionals to organise protests in the nude (cases 1-3) on the issue of heightened vulnerability to contracting the coronavirus in the context of acute shortages of personal protective equipment. Furthermore, social media platforms (e.g. Twitter or Facebook) that allow simultaneous sharing of different types of online material (e.g. video, photograph, file, or text) broaden the appeal of the material to the intended audience. In the cases analysed, the social media material and how these contributions were received online also attracted the attention of traditional news outlets whether online or offline. Thus, press coverage had raised the online profile of the authors notwithstanding the likely reputational effects.

As the number of social media cases analysed is relatively small, the findings from this exploratory study provide limited insight into the motivations of doctors and dentists in their social media contributions during the early days of the Covid-19 pandemic. Further research would be undertaken in the future with a more systematic approach. However, the recurring themes informed by the findings are useful in helping to develop some understanding as to what influenced doctors and dentists to create social media contents that might not lend well to the standards expected by professional regulators.

On the first issue of practitioners utilising social media to draw attention to lapses of PPE or to adverse issues in clinical workplace, it is interesting to observe that whistleblowing is a rather grey area for both the medical and dental professions. Notably, the GMC has not made its position on whistleblowing via social media known in its guidance, though it stated elsewhere that a professional duty of candour ought to be exercised.^40^ The idea behind this is that a duty of candour would increase disclosure of concerns and that whistleblowing would become commonplace.^41^ This would in effect create a transparent healthcare environment conducive to better standards of patient care.^42^ The GMC nevertheless emphasised that such concerns, in particular relating to patient safety, ought to be escalated via formal organisational processes established in the workplace.^43^ Appreciably, though there is an imperative to raise concerns in relation to healthcare delivery, it is apparent that whistleblowing via a social media platform might not therefore be wholly appropriate. On a similar note, the GDC reminds its practitioners of the responsibility to raise concerns especially in circumstances where dental practices may place patients or colleagues at risk. The manner in which whistleblowing occurs is an important factor to the GDC. Unlike the GMC, it has explicitly advised in its social media guidance that such platforms should not be used for such intentions. Thus, social media interventions intended to draw attention to whatever professional or workplace-related issues would contravene these guidelines. This is even more so in the case of clinicians who create contents with pictures of nudity which are then subsequently shared with the public on social media. Not only do these fall foul of the prohibition on whistleblowing, but judging by previous incidents of healthcare professionals who had posted ‘inappropriate’ photographs about themselves, they would constitute unprofessional conduct which have the potential to bring their professions into disrepute.^44^ As a consequence, these actions may actually undermine public confidence in the profession irrespective of whether these actions are motivated by a simple desire to bring about improvements to healthcare or by the professional duty of candour.

On the second issue where healthcare professionals make online interventions with the effect of discrediting their own regulatory bodies, both the GMC and GDC affirm that it is considered unprofessional for medical and dental professionals to proffer comments, opinions, or remarks that are of a denigrating nature against fellow professionals. It was stressed that they must treat one another in a respectful manner,^45^ and that communication through social media is not entirely different in kind from any other verbal or written communications.^46^ Moreover, commentary involving specific individuals or organisations are subject to defamation laws.^47^ Consequently, all forms of written or verbal communications made in a personal or professional capacity may be the subject of legal action.^48^ Even if made in good faith and with the intention of drawing attention to what the complainant may perceive to be genuine concerns, both regulators believe that social media ought not be an appropriate means for raising professional concerns. By the same token, social media should not be utilised to organise online campaigns against specific individuals or organisations. Therefore, online personal attacks and protests are likely to be deemed as professional misconduct.^49^ Moreover, the GDC makes clear that *sharing* disrespectful comments, as distinct from *making* disrespectful comments,^50^ via social media is also likely to amount to professional misconduct. Hence, individuals who had shared and ‘liked’ comments denigrating the regulator too may fall foul of the regulator’s expectations on the standards of social media usage.^51^

The third issue that has been raised by this study concerned social media material containing information that were later found to be examples of misrepresentation, misinformation, and unqualified professional opinions. Here, it is important to emphasise that the GMC takes the view that any material created by authors who represent themselves as medical professionals are likely to be taken on trust by the lay readership.^52^ The GMC believes that the general public may take professional views espoused by individual doctors to be representative of the views of the profession overall. Interestingly, the GDC does not hold an explicit view on this matter. While doctors are expected to disclose their true professional identities online and are advised against making or sharing online content anonymously, dentists are under no regulatory obligation in this regard. Dentists are nevertheless advised by the GDC to ‘think and consider carefully’ the potential impact of their actions. When considered alongside the warnings given by both the GMC and GDC to avoid making factually inaccurate or unsubstantiated opinions online, doctors and dentists are expected to personally exercise due diligence *and* to uphold patient confidentiality at all times. Therefore, doctors and dentists must refrain from expressing professional opinions that are outside the scope of their usual clinical practice. Professional sanctions and disciplinary action may be pursued against those who create social media content that are later found to be untrue or have been made without due care. In view of this, social media material created by doctors and dentists found to be sources of public misinformation will have contravened GMC and GDC social media guidelines had the authors concerned been UK healthcare professionals.^32^

## Conclusion

During the Covid-19 global pandemic, many medical and dental practitioners are utilising social media for information sharing, professional networking, engaging with the public and patients, and for training and educational purposes. However, some from these two professional groups have also used this platform to express their health and safety concerns directed towards parties like the government and their employers; as well as to vent their frustrations against their regulators; and to proffer their professional views in ways which can affect public opinion about the pandemic. Given the wide-reaching scope and the ability of social media to disseminate information rapidly, it is not difficult to see why medical and dental professionals have resorted to this medium of communication as their outlet. However, as members of professional bodies, they do not ordinarily and necessarily enjoy the same liberties the general public have in the manner and nature of their online posts. In addition to the need to ensure that they do not contravene the law, they are also usually guided, warned, and constrained by the guidelines issued by their regulatory bodies. Failure to adhere to such guidance may amount to professional misconduct that could be subject to a formal disciplinary investigation. Importantly, the regulatory bar for social media misconduct is set lower than the criminal bar determined by the law.^53^

This article set out to assess whether sensationalist online posts relating to Covid-19 contravene existing GMC and GDC social media guidelines. Three broad themes were identified namely whistleblowing; discrediting regulators; and misinforming the public. The study deduced that had all these materials been created by equivalent professionals in the UK, it is highly likely that the authors concerned would come into conflict with the regulatory warnings and cautions pertaining to: whistleblowing via an inappropriate forum; making unsavoury comments relating to fellow professionals including those in positions of leadership; or offering professional opinion without paying sufficient heed to their veracity and/or wider impact. As the online activities reviewed in this study are, on the face of it, unprofessional behaviours, UK-based practitioners would do well not to be swayed by the public’s comparative freedom when using social media during the pandemic. Any doctor or dentist found not adhering to the current social media guidance may risk being disciplined once the dust has settled on Covid-19.

However, in the absence of additional regulatory guidance on online professionalism and on social media conduct in the context of the pandemic,^54^ should there be concessions^55^ allowed given the extraordinary and extenuating circumstances posed by Covid-19? Further, should the effect of the current pandemic be allowed to empower and encourage healthcare professionals to test the likely regulatory boundaries when engaging with social media? In any event, would public health sentiments raised this way still attract perceptions from the general public that the actions of those medical and dental clinicians had placed the respective professions into disrepute? As is widely appreciated, the rapidly evolving and dynamic nature of the Covid-19 public health emergency has created multiple socio-economic pressures,^56^ medico-legal dilemmas,^57^ and ethical concerns.^58^ If healthcare professionals through their contributions on social media identify and raise public awareness of lapses in healthcare provision, patient care, or public safety, then can it be surmised that such actions garner public support irrespective of the manner through which the issues were raised? In these circumstances, would regulatory bodies be circumspect in their sanctioning of clinicians with their disciplinary procedures?

At the same time, given that health information via social media have the potential to conflict with one another and create confusion for the online public audience, doctors and dentists ought to be mindful that their messages and commentaries are likely to influence the choices and decisions of their lay readership. After all, healthcare professionals are not only perceived to be important sources of reference and information, but also considered as trusted and reliable sources. However, this should be considered with the caveats that (a) inaccurate, conflicting, and unsubstantiated online contents regularly percolate through social media;^59^ and (b) the processes of monitoring social media material and their quality assurance are unclear.^60^ In view of the evolving nature of how public health messages reach the general population and on the inconsistencies in the advice given by governments globally,^61^ should there therefore be an imperative for clinicians to engage with social media more, albeit in a cautious manner?

On the whole, increased utilisation of social media by healthcare professionals are indicative of shifting practices and should be welcomed as technological progress. However, the concept of online healthcare professionalism, what it means, and its likely principles for online practice require further development. One lesson on social media engagement that may be taken away from the experience of this Covid-19 pandemic, is that social media offer healthcare professionals the opportunity and space to make their individual contribution to tackling the crisis, yet at the same time presenting them with various ethical and professional pitfalls that they should be mindful of.

In relation to the professional regulators, although the GMC and GDC issued their social media guidelines in response to increased social media usage amongst healthcare professionals, technology has advanced since these were issued. What is equally important to note is that those guidelines were issued during peacetime, so to speak. Is it fair to apply such guidance without an appreciation of the public health emergency posed by Covid-19? Although broadly similar, the guidance prescribed by the GMC and the GDC contrast in their emphases on online professionalism and diverge somewhat on how an individual healthcare professional should conduct themselves through social media. Since there are increasing numbers of doctors and dentists utilising social media to make their voices heard, the existing guidance issued by the two regulatory bodies clearly need to remain relevant with changing times. Covid-19 presents an opportunity to update, clarify, and align their social media advice with seemingly fast-moving times.
